# Thermotolerant isolates of *Beauveria bassiana* as potential control agent of insect pest in subtropical climates

**DOI:** 10.1371/journal.pone.0211457

**Published:** 2019-02-01

**Authors:** Sumer Alali, Valeria Mereghetti, Franco Faoro, Stefano Bocchi, Fawaz Al Azmeh, Matteo Montagna

**Affiliations:** 1 Dipartimento di Scienze Agrarie e Ambientali, Università degli Studi di Milano, Milan, Italy; 2 National Commission for Biotechnology, Damascus, Syria; 3 Dipartimento di Scienze e Politiche Ambientali, Università degli Studi di Milano, Milan, Italy; Chinese Academy of Agricultural Sciences Institute of Plant Protection, CHINA

## Abstract

The use of *Beauveria bassiana* in biological control of agricultural pests is mainly hampered by environmental factors, such as elevated temperatures and low humidity. These limitations, further amplified in a global warming scenario, could nullify biological control strategies based on this fungus. The identification of thermotolerant *B*. *bassiana* isolates represents a possible strategy to overcome this problem. In this study, in order to maximize the probability in the isolation of thermotolerant *B*. *bassiana*, soil samples and infected insects were collected in warm areas of Syria. The obtained fungal isolates were tested for different biological parameters (i.e., growth rate, sporulation and spore germination) at growing temperatures ranging from 20°C to 35°C. Among these isolates (eight from insects and 11 from soil samples), the five with the highest growth rate, spore production and germination at 30°C were tested for their entomopathogenicity through *in vivo* assays on *Ephestia kuehniella* larvae. Insect mortality induced by the five isolates ranged from 31% to 100%. Two isolates, one from *Phyllognathus excavatus* and one from soil, caused 50% of the larval mortality in less than four days, reaching values exceeding 92% in ten days. These two isolates were molecularly identified as *B*. *bassiana* sensu stricto by using three markers (i.e., ITS, *Bloc* and *EF1-α*). Considering these promising results, further studies are ongoing, testing their efficiency in field conditions as control agents for agricultural insect pests in Mediterranean and Subtropical regions.

## Introduction

Global climate change poses new challenges to agricultural production [1], increasing crops’ vulnerability to plant diseases and pests mainly as a consequence of physiological stresses [2], and the expansion of insects and diseases into higher latitudes [3]. The introduction of new pests, aside from endangering food security, requires the adoption of new strategies for their control. This phenomenon particularly affects temperate countries, where the expansion of species range due to climate change has been well documented [4]. In particular, it has been estimated that insects and diseases are expanding towards the poles at 7 km per year [5]. In agriculture, adaptive strategies have already been used to improve the capability of plants to face increasing abiotic stresses and to optimize growth and phenology in relation to new seasonal variations [6–7]. In pest control, the keystone adaptive strategy relies on integrated pest management (IPM), which depends upon different integrated practices starting from an accurate estimation of the pest abundance based on monitoring and working towards the adoption of biological control strategies to reduce of conventional pesticide application [8]. Mycoinsecticides, mainly consisting of the fungal pathogen *Metarhizium anisopliae* (Metschn.) Sorokın and *Beauveria bassiana* Balsam are a fundamental component of these biological control strategies [9,10]. Even though these fungi have already been successfully adopted in large-scale applications (e.g., for the control of the Sahelian grasshopper *Oedaleus senegalensis* Krauss, 1877 [11] and the Masson’s pine caterpillar *Dendrolimus punctatus* Walker, 1855 [12]) and their use is increasing yearly (20–44%) in Europe and North America [13], they suffer major limitations. In fact, different abiotic factors, such as UV radiation, low humidity and high temperature, hamper fungal biological activity and, in turn, their effectiveness as biocontrol agents [14–15]. For example, the optimal growth temperature of *B*. *bassiana* is between 25°C and 28°C [16], though it has been demonstrated that some isolates are able to grow at a higher temperature (~30°C) with highly reduced activity and may not survive at 34°C [17,18]. To overcome this limitation, a possible strategy has been developed, which relies on genetic improvement of the fungus in terms of its tolerance to oxidative stress was developed [19,20]; however, this approach suffers regulatory and societal limitations related to the use of genetically modified organisms [21]. A possible alternative strategy consists of mass screening of fungal isolates for the target phenotype, a successful strategy in the case of UV tolerance [22].

**Fig 1:**
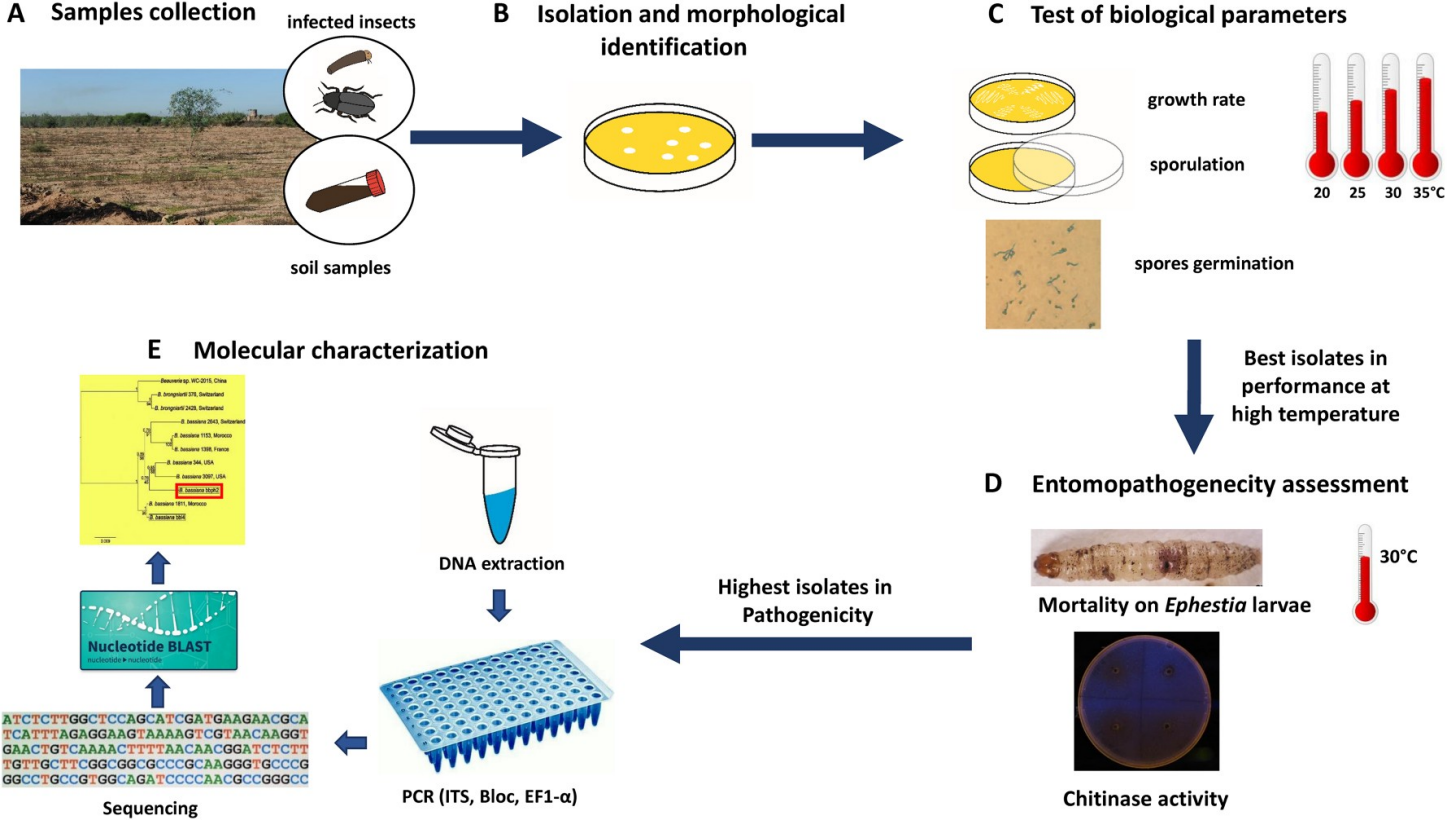
Selective process adopted to isolate thermotolerant *Beauveria bassiana*. (A) Collection of infected insects and soil samples from hot areas. (B) Isolation and morphological identification of the obtained isolates. (C) Biological traits tested at different temperatures (20°C, 25°C, 30°C and 35°C). (D) Selection of the best isolates in performance and entomopathogenicity evaluation at 30°C. (E) Molecular identification of the most aggressive isolates.

In this study, in order to overcome temperature limitations in the use of *B*. *bassiana* as a biocontrol agent, *ad hoc* selective scheme (Fig 1) was adopted with the aim of finding out effective thermotolerant *B*. *bassiana* isolates, starting with infected insects and soil samples collected in different areas of Syria, subjected to a high environmental temperature.

## Materials and methods

### Ethics statement

The study was carried out on private lands with the permission of the owners; none of the collected species are listed in national or regional law as protected or endangered.

### Fungal isolates

**Fig 2:**
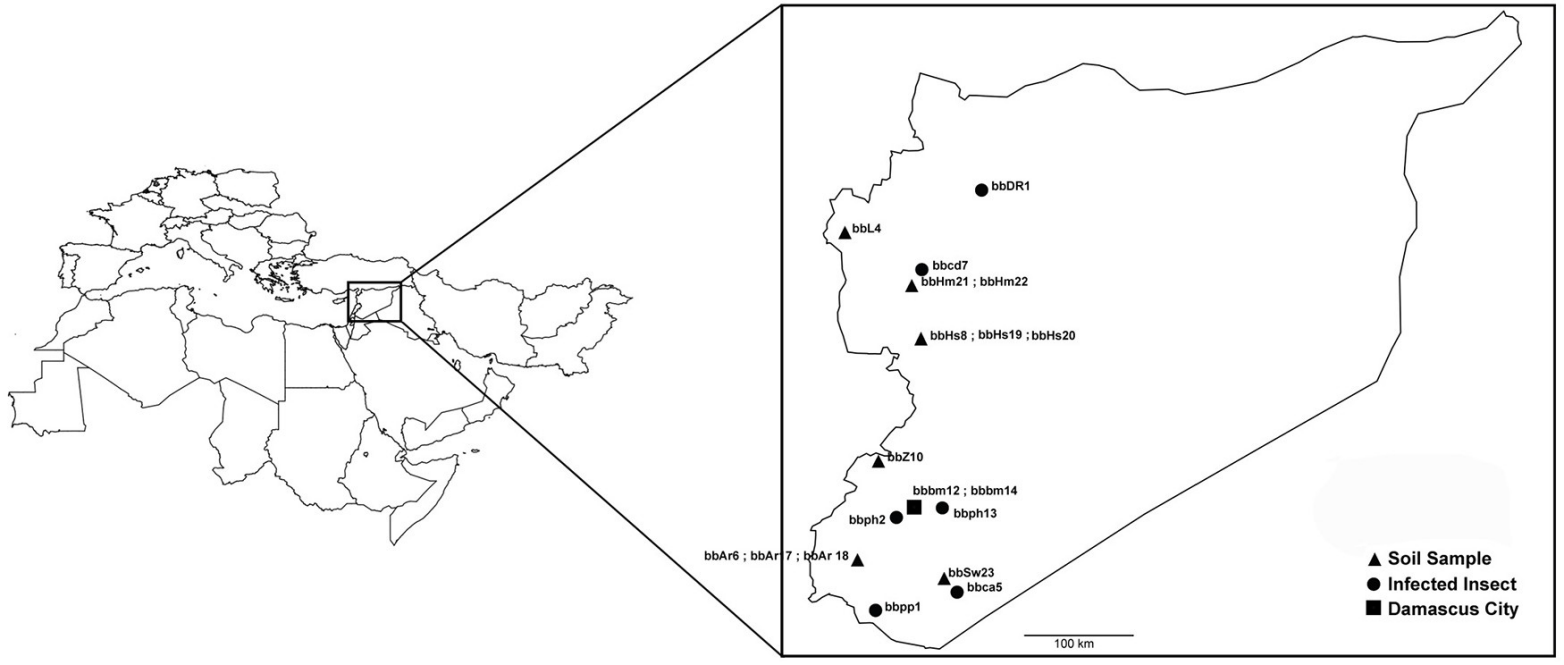
Geographical location of the area where the samples were collected. The inset shows the sampling sites distribution within the Syrian borders, the origin of the sample (soil or infected insects) and the code of the isolates. Maps were plotted by using the function *map* in the R package “maps” [28].

Fungi were isolated from insect specimens showing superficial hyphal growth and from soil samples, both collected directly from fields in different hot Syria regions (S1 Table; Fig 2). Isolation from soil samples (five replicates each) was performed by the insect trap method [23], modified by using 4-5^th^ instar larvae of *Ephestia kuehniella* (obtained from insectary at the Biological Control Studies and Research Centre, Damascus University-Faculty of Agriculture). *E*. *kuehniella* larvae were observed every week up to three weeks and those with evident fungal growth were selected. Infected insects (i.e., insect collected from the field and *E*. *kuehniella* larvae experimentally infected), were surface sterilized and incubated in a moist chamber up to the appearance of an actively growing mycelium [24]. This mycelium was transferred to SDYA [25]. After two weeks, fungal isolates were subcultured using a selective medium (40 g glucose, 10 g proteose peptone, 15 g agar, 0.01 g crystal violet, 0.25 g cyclohexamide, 0.5 g chloramphenicol, 1L distilled water) [26]. Isolates were subcultured several times to ensure purity and monosporic cultures from all isolates were obtained, then morphologically identified as *B*. *bassiana* s.l. by a microscope Olympus BX51 (Olympus, Japan) at 400x magnifications and the morphological key [27]. The selected isolates are stored in the microorganisms repository of the Dipartimento di Scienze Agrarie e Ambientali, Università degli Studi di Milano—Italy.

### Spores preparation for further assays

The obtained isolates were cultured on SDYA medium plates at 24±1°C for 14 days. Fresh conidia were harvested from the plates by adding 10 ml of 0.05% Tween 80, and then transferred to a sterile plastic tube (50 ml), vortexed for 3 min and filtered through one layer of sterilized filter paper (Watmann n. 01). Conidial concentration was calculated by using a hemocytometer and adjusted to 10^6^ spores × ml^-1^ by dilution to be used in the following assay [29].

### Biological traits of *Beauveria bassiana* isolates

Biological traits of isolates, growth rate, sporulation and spores’ germination were estimated on SDYA medium plates at four different constant temperatures: 20±0.1°C, 25±0.1°C, 30±0.1°C and 35±0.1°C. The growth rate and spore production were estimated according to the method described by Mustafa and Kaur [30]. Five replicates of each isolate were prepared. The growth rate of the colony was obtained measuring its diameter after 14 days. At the same time, five pieces of the grown mycelium (1 cm^2^) were randomly taken from the colony; samples were placed in 5 ml of 0.05% Tween 80 solution and vortexed. Spore yield was determined using a haemocytometer.

Spore’s germination was determined by the agar slide technique [30]. Media coated slides were inoculated with 100 μl of spores’ suspension (10^6^ spores × ml^-1^) from each isolate. The inoculated slides (5 for each isolate) were placed in Petri dishes and incubated at the selected temperatures (i.e., 20±0.1°C, 25±0.1°C, 30±0.1°C and 35±0.1°C). Slides were observed by a light microscope (400×) after 24 hours. A conidium was considered germinated when a distinct germ tube of length twice its diameter was observed. In order to obtain reliable estimates of germination percentage [31], 300 conidia were scored for each replicate. Isolates with the best performance at high temperature were selected to perform the entomopathogenicity assays.

Fungal growth rate (mm/day), conidial production (conidia/ml) and spore germination ratio (number of germinated spores/300 spores), expressed as percentage, were analysed as completely randomized design using a one-way ANOVA and Tukey test for post-Hoc analysis by using (SPSS.18). In addition, the percentage of the difference in performance due to the increase of temperature from 25°C to 30°C were calculated for each biological trait [32], and its correlation with the hot season’s average temperature of the sites of sampling [33], were tested by Pearson coefficient by using (SPSS.18).

### Evaluation of entomopathogenicity

The evaluation of isolates entomopathogenicity was tested in laboratory on *E*. *kuehniella* 3^rd^ instar larvae after surface sterilization as in Goettel and Inglis [15]. For each isolate, five concentrations of conidia were tested (from 1×10^2 to^ 1×10^6^, spores/ml). Larvae were dipped singly into the suspension at the tested concentration for 1s, and then moved to a sterile filter paper to dry. Three replicates of each isolate for the tested concentration were incubated in a humid chamber at 30±1°C; each replicate consists of ten treated larvae. The control was prepared by dipping larvae into 0.05% Tween 80 solution. Every two days, over a period of ten, larvae were observed in order to record the mortality and the symptoms used to estimate the Fungal Development Index (FDI [34]) (S2 Table). In addition, the corrected mortality [35], LC_50_ and LT_50_ were calculated by using Probit analysis in SPSS 18.

As it is well known that *B*. *bassiana* entomopathogenicity is correlated with the production of an array of cuticle-degrading extracellular enzymes, chitinase plate assays were performed [30]. The enzymatic activity index was calculated using the formula of St. Leger et al [36].

$$\text{enzymatic}\;\text{activity}\;\text{index}\;=\;\frac{\text{Ø}\;\text{halo}+\text{Ø}\;\text{colony}}{\text{Ø}\;\text{colony}}$$

### Molecular characterization

In order to confirm the previous morphological identification, isolates with the highest entomopathogenic activity at high temperature were molecularly characterized. DNA was extracted from mycelium grown in SDY broth [37]. DNA was extracted from 200 μg of dried mycelium using the CTAB method described by Möller [38] with the modifications of Safavi [39]. Three nuclear markers were used to perform the molecular characterization of the selected isolates according to previous studies [40,41]. The selected markers were: i) the internal transcriber spacers I, II and the ribosomal 5.8S rRNA (here after ITS); ii) the B locus intergenic region (*Bloc*), and iii) the translation elongation factor 1 alpha (*EF1-α*). The used primers and PCR amplification conditions are reported in S3 Table.

Successful amplifications were determined by gel electrophoresis and PCR products were bidirectionally sequenced by ABI technology (Applied Biosystems, Foster City, CA, USA). The obtained electropherograms were manually edited and assembled into a consensus sequence using Geneious Pro 5.3 (Biomatters Ltd., Auckland, New Zealand); the consensus sequences were deposited in ENA archive (accession numbers: LT903665, LT903666, LT903693-LT903696). Sequences were identified by using BlastN [42]. In order to perform the phylogenetic analyses of the selected isolated of *B*. *bassiana*, orthologous sequences of the selected markers were collected from GenBank (S4 Table). Orthologous sequences of *Beauveria* sp. and *Beauveria brongniartii* were included in the dataset as outgroups (S4 Table). The obtained dataset, one for each locus, were aligned using MAFFT [43], with G-INS-i as the search strategy [44]. The nucleotide substitution models were estimated for the three data sets using jModelTest 2 [45] and selected according to the Akaike information criterion (AIC). The analyses selected the GTR [46] with proportion of invariable sites (I) as the best model for the *EF1-α* dataset; the HKY [47] with I and site-heterogeneity in evolutionary rates (Γ-distribution parameter) as the best for *Bloc* and ITS dataset. Phylogenetic inference was performed, using both Maximum Likelihood and Bayesian Inference approaches, on single-marker and on the dataset obtained after their concatenation. Maximum likelihood analyses were performed using PhyML version 3.0 [48] with the best of NNI and SPR as tree searching operation and node support estimated using 100 bootstrap replicates. Bayesian analyses were performed using MrBayes 3.2 [49] in two independent runs, with one cold and five heated Markov chains (λ = 0.1) each, for 2 × 10^6^ generations, that were sampled every 100 generations. The convergence of each run was visually inspected using TRACER [50] and an appropriate number of trees discarded as burn-in.

## Results

### Identification of fungal isolates

**Fig 3:**
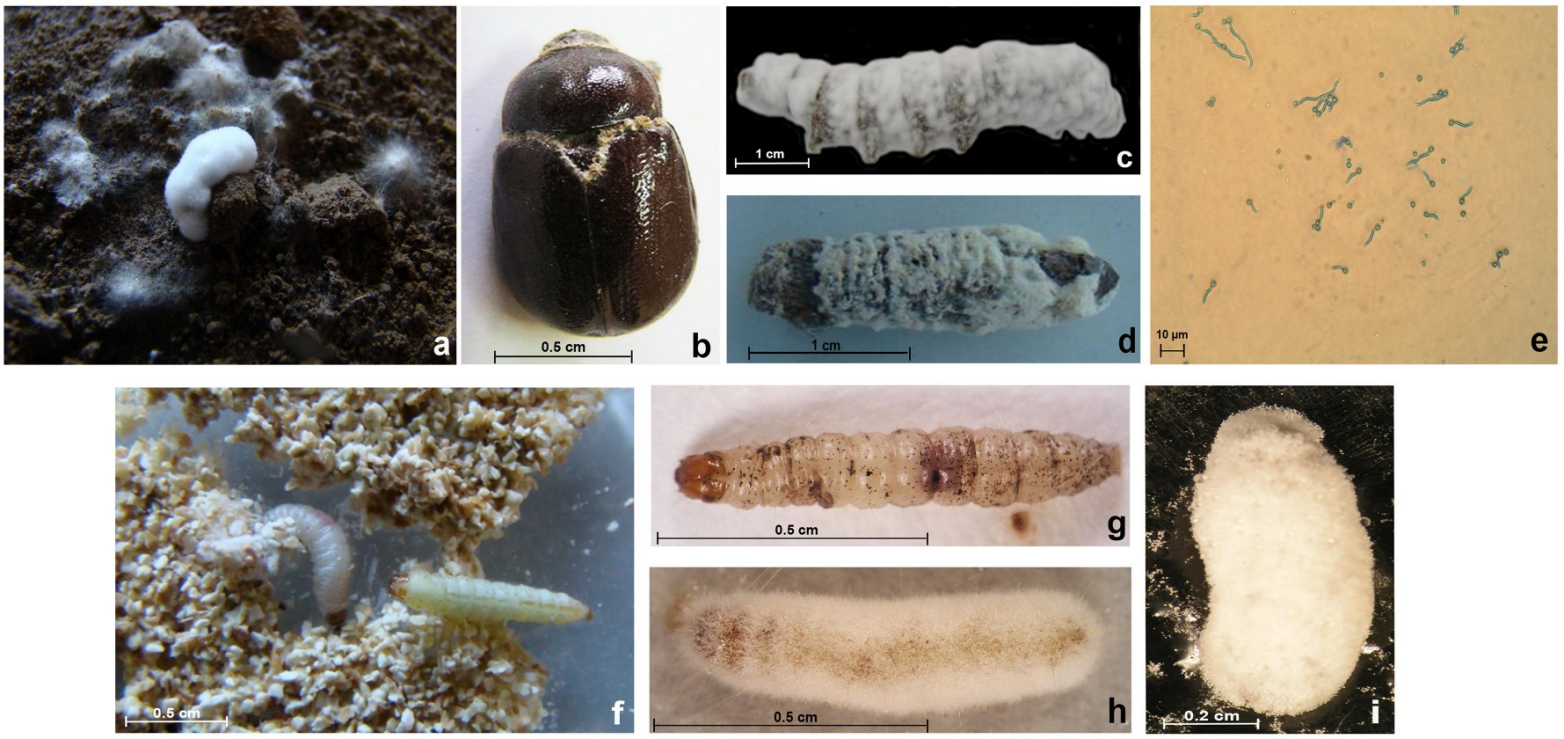
The collected insects infested by *Beauveria bassiana*, with the degrees of FDI on *Ephestia kuehienella* larvae. (a) infected larva of *Ephestia kuehniella* in soil trap method; (b) *Phyllognathus excavatus* adult (♀) infected with *Beauveria bassiana*; (c) *Bombyx mori* larva infected with *B*. *bassiana*; (d) *Paropta paradoxa* larva infected with *B*. *bassiana*; (e) Germination of *B*. *bassiana* spores; (f) Healthy larvae of *E*. *kuehienella* (3^rd^ instar); (g) larva of *E*. *kuehienella* with milanic spots (FDI = 0.5); (h) dead larva of *E*. *kuehienella* covered with mycelium growth (FDI = 2); (i) larva of *E*. *kuehienella* covered with *B*. *bassiana* growth in full sporulation (FDI = 3).

A total of 19 isolates were morphologically identified as *B*. *bassiana*. Isolates were retrieved from both insects (eight isolates) and soil samples (11 isolates) (S1 Table; Fig 3a). The insect hosts were adults of *Phyllognathus excavates* (Fig 3b), *Capnodis* spp., *Cerambyx dux* and *Eurygaster integriceps*, and the larvae of *B*. *mori* (Fig 3c) and *Paropta paradoxa* (Fig 3d).

### Biological traits of *Beauveria bassiana* isolates

Isolates were significantly different in their responses to temperature in terms of biological traits. In general, all isolates showed their highest biological performances at 25°C. Isolates obtained from areas with lower temperatures were superior in growth rate (e.g., *bbca5* with 2.24±0.12 mm × day^-1^), whilst those collected from the hottest areas had the lowest values (e.g., isolate *bbbm12* with 1.71±0.08 mm×day^-1^) (S5 Table). At 25°C, the highest spore germination was recorded for *bbL4* (3.46±0.05 spores × ml^-1^), whilst the lowest value was recorded for isolate *bbSw23* (1.19±0.03 spores × ml^-1^); spore germination ≥ 96% occurred for all isolates except *bbDR1* (92.7±0.24%) (S5 Table; Fig 3e).

With a growth temperature of 30°C, isolates obtained from samples collected in the hottest areas showed the highest values of measured biological traits (i.e., growth rate, spore production and spore germination). However, at this temperature the obtained values were lower than those achieved at 25°C. For all the tested traits, five out of the 19 isolates were superior (S5 Table), since they suffered a limited decrease in performance due to the increase of temperature from 25°C to 30°C (S6 Table). In addition, significant negative correlations were found between the reduction of tested biological parameters (growth at 25°C and 30°C, see Material and methods) and the average temperature of the hot season at the isolates’ collecting sites (Pearson coefficient (r) = -0.56, -0.45 and -0.54 for growth rate, spore production and spore germination reduction (%), respectively; P-value < 0.001) (S6 Table). At a growth temperature of 20°C, all the biological trait values were reduced in respect of those obtained at 25°C and 30°C (S5 Table), whilst no growth was recorded at 35°C. At this extreme temperature, only 2% of the isolates were able to germinate, but the germination tube soon aborted.

In view of the above results, isolates *bbph2*, *bbph13*, *bbL4*, *bbbm14* and *bbpp1* were selected for the bioassays for their pathogenicity on larvae of *E*. *kuehniella*.

#### Entomopathogenic activity assessment

The pathogenicity of the five selected *B*. *bassiana* isolates (i.e., *bbpp1*, *bbph2*, *bbL4*, *bbph13* and *bbbm14*; S5 Table) was evaluated on the insect model *E*. *kuehniella* at 30°C. At a spores’ concentration of 10^2^ and 10^3^, no mortality was recorded after ten days for all the selected isolates (Fig 3f). The FDI was 0 in the case of the first spore concentration, but when 10^3^ spores were inoculated, small melanic spots appeared on some larvae (0.5 ≥ FDI > 0; Table 1; Fig 3g). At a spore concentration of 10^4^, the mortality arose up to 26% in the case of the bioassays with *bbL4* and *bbph2* isolates; however, no mortality was recorded with *bbph13* and *bbbm14* isolates. The increased mortality was accompanied by a significant difference in FDI values between the isolates; the highest values were recorded for *bbL4* and *bbph2* (FDI_*bbL4*_ = 0.9, FDI_*bbph2*_ = 0.83), indicating the presence of large melanic spots on most of the larvae (Fig 3g). At a spore concentration of 10^5^, the mortality reached 100% in the case of isolate *bbph2*, followed by isolate *bbL4* (93%). The two isolates *bbpp1* and *bbbm14* caused mortality lower than 50% of the *E*. *kuehniella* larvae. At the highest tested spore concentration (i.e., 10^6^), isolates *bbph13*, *bbph2* and *bbL4* caused a larvae mortality of 100% vs. less than 70% in the case of *bbpp1* and *bbbm14*, with an FDI of 1.57 and of 1.53, respectively (Fig 3h and 3i).

**Table 1:** Mortality of the larvae and fungal development index at the 10^th^ day of treatment.

Isolate	10^2^^a^		10^3^		10^4^		10^5^		10^6^	
	M	FDI	M	FDI	M	FDI	M	FDI	M	FDI
*bbL4*	0	0	0	0.23±0.07	26.67±6.67*	0.9±0.1*	93.1±6.9*	2.73±0.27*	100±0*	2.9±0.06*
*bbph2*	0	0	0	0.1±0.06	26.67±6.67*	0.83±0.09*	100±0*	2.87±0.03*	100±0*	2.95±0.03*
*bbph13*	0	0	0	0.27±0.07	0±0	0.58±0.04	75.8±0	1.65±0.1	100±0*	2.9±0.03*
*bbbm14*	0	0	0	0.22±0.07	0±0	0.37±0.02	31.03±6.9	1.02±0.1	67.86±6.19	1.53±0.04
*bbpp1*	0	0	0	0.05±0.03	13.33±3.33	0.43±0.04	48.28±0	1.43±0.02	51.72±3.45	1.57±0.09
F ^b^				2.46	9.067	17.42	18.542	16.22	9.096	14.5
Sig				0.080	0.000	0.000	0.000	0.000	0.000	0.000

M, corrected mortality (%) reported in (mean ± SE); FDI, fungal development index reported in (mean ± SE).

^a^ concentration of the inoculum as spores/ml;

^b^calculated Fisher’s test value;

*in the same column indicate the significantly higher values (α = 0.05).

Estimation of the LC_50_ showed that isolates *bbph2* and *bbL4* had the lowest values, with 1.65×10^4^ spores/ml and 2.01×10^4^ spores/ml, respectively (Table 2, S1 and S2 Figs). For these two isolates, the LT_50_ values, at a spore concentration of 10^5^, were estimated to be 3.85 and 3.66 days, indicating their higher level of aggressiveness (Table 2).

**Table 2:** Aggressivity assessment of selected isolates of *Beauveria bassiana*.

Isolates	LC_50_^a^	LT_50_^b^	Chitinase Activity Index (mean ± SE)
	at 10^th^ day	at 10^5^	
*bbph2*	1.65 × 10^4^	3.85	1.35±0.05*
*bbL4*	2.01 × 10^4^	3.66	1.31±0.02*
*bbph13*	5.74 ×10^4^	8.97	1.26±0.06
*bbbm14*	35.82 × 10^4^	12.10	1.22±0.05
*bbpp1*	37.72 × 10^4^	10.42	1.23±0.03
F^c^			4.222 (*p-value* = 0.001)

^a^LC_50_ measured by spore × ml^-1^;

^b^LT_50_ measured by days;

^c^calculated Fisher’s test value;

*in the same column indicate the significantly higher values (α = 0.05).

All five selected isolates showed chitinase activity. However, a difference between the isolates was recorded. Isolates *bbph2* and *bbL4*, with a 1.35 and 1.31 chitinase activity index, showed the highest values (Table 2).

### Molecular characterization

In order to perform the molecular characterization of *bbph2* and *bbL4* isolates (with the highest biological characterization scores and the highest virulence), three nuclear markers were amplified through PCRs. All three markers (i.e., ITS, *Bloc* and *EF1-α*) led to positive amplifications and sequencing for both isolates. The obtained consensus sequences were subjected to BLAST analyses: ITS and *Bloc* sequences (~550 bp and ~1440 bp, respectively) of both isolates possessed 100% of sequence identity with the orthologous sequences of *B*. *bassiana* (ITS: isolate ArgB10, accession number KT378227; strain MTCC_6286, accession number JQ266208; *Bloc*: strain RCEF5438, accession number JQ867110; strain ARSEF 1040, accession number HQ880689). Instead, *EF1-α* amplicons (~950 bp) possessed 99% of identity with *B*. *bassiana* isolate KVL 03_90 and strain ARSEF 1554 (accession numbers EF193191 and AY883693, respectively).

**Fig 4:**
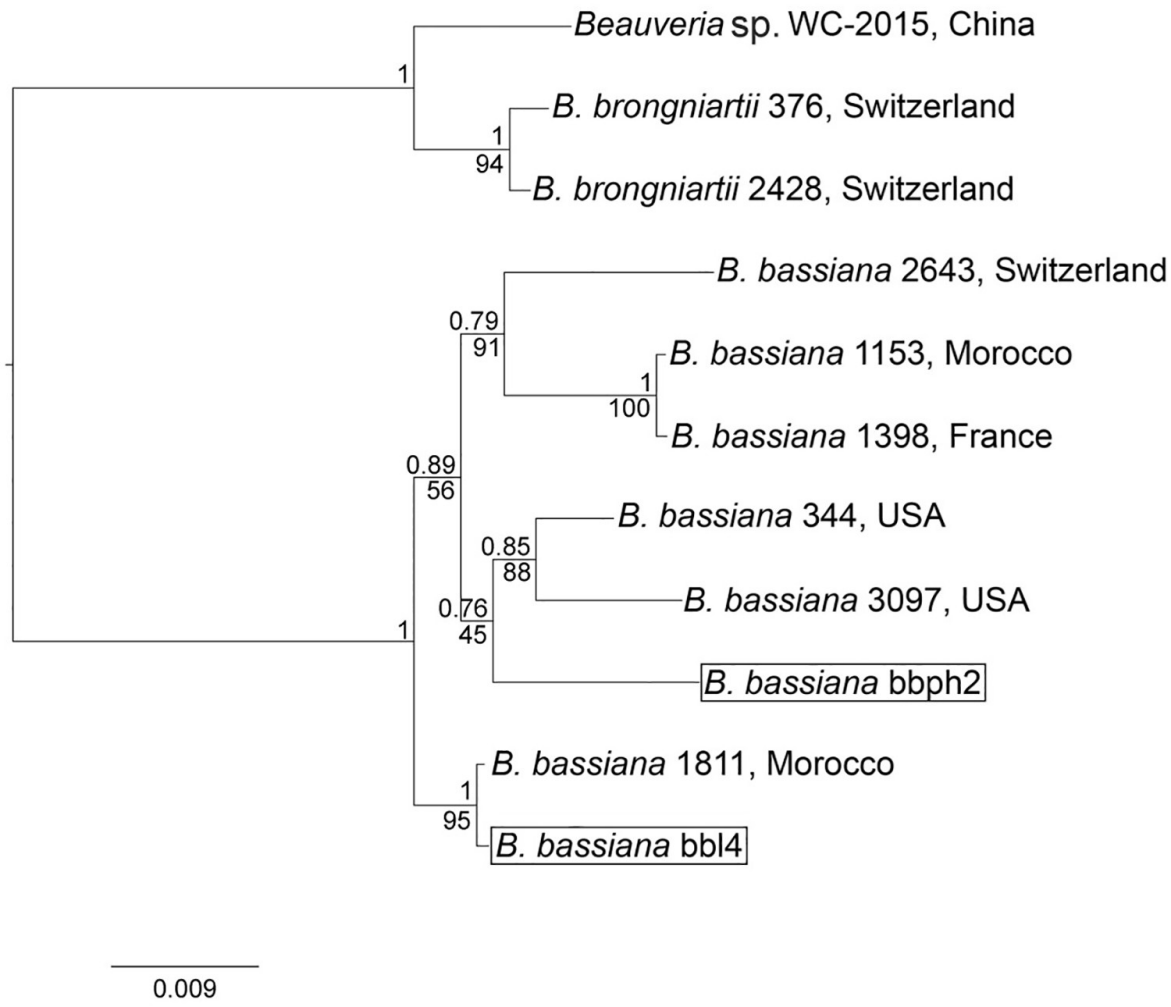
Bayesian consensus tree inferred on ITS, *Bloc* and *EF1-α* gene sequences. Support values expressed as Bayesian posterior probability are reported above the nodes, while below each nodes the approximate likelihood ratio test (as obtained in the Maximum Likelihood phylogenetic inference analysis performed on the same dataset) are recorded. Outgroups: *Beauveria* sp. and *Beauveria brongniartii*. The scale bar at the bottom indicates the distance expressed in substitutions per site. Isolates obtained in this study are surrounded by black lines.

Phylogenetic reconstructions, with both maximum likelihood and Bayesian inference approaches, were performed for each single-gene dataset as well as for the dataset obtained concatenating the three gene sequence alignments (Fig 4; S3, S4 and S5 Figs). All the obtained topologies are in agreement and confirm, with a high node support (Bayesian posterior probability = 1 and bootstrap = 100), the phylogenetic position of *bbph2* and *bbL4* isolates in the *B*. *bassiana* clade (Fig 4). In details, *bbL4* isolates resulted sister of *B*. *bassiana* ARSEF 1811 [41], isolated from the weevil *Sitona discoideus*, collected in Morocco. Isolate *bbph2* clustered within a clade composed of *B*. *bassiana* ARSEF 344 and ARSEF 3097, respectively, isolated from the leaf beetle *Leptinotarsa decemlineata* and from the weevil *Anthonomus grandis*, collected in the USA [41].

## Discussion

In this study, all *B*. *bassiana* isolates germinated and grew at 20°C, 25°C and 30°C, but not at 35°C, as expected. Five *B*. *bassiana* isolates, namely *bbpp1*, *bbph2*, *bbL4*, *bbph13* and *bbbm14*, performed well in the tested biological traits, also growing at 30°C (S5 Table). It is worth noting that four out of the five isolates were collected in the hottest areas. The only exception is represented by isolate *bbL4*, obtained from a site with a lower temperature (27°C). The results of this study support the existence of a relationship between the termotholerance ability of the isolates and their geographical origin, even if this is still under debate [32,51]. In terms of pathogenicity, the five selected isolates induced a differential mortality on the model insect (i.e., *E*. *kuehniella*), with values ranging from 31% to 100% (Table 1). Isolates *bbph2*, *bbL4 and bbph13* achieved more than 75% mortality, whereas isolates *bbph2* and *bbL4* showed the highest values of FDI within the same time period (2.73±0.27, 2.87±0.03, respectively), which highlights the difference in pathogenicity levels between the isolates [52]. The achieved values of pathogenicity further support adaptation of the isolates to growth at a relatively high temperature. In addition, the isolates *bbph2* and *bbL4* possessed a low value of LC_50_ (1.65 × 10^4^ and 2.01 × 10^4^, respectively), which are in a range of values considered effective on lepidopteran larvae as *E*. *kuehniella* and *Plodia interpunctella* [53,54]. The values of LT_50_ obtained for these two isolates (less than four days) confirm their aggressivity, according with the findings of Sosa-Gomez [55]. In addition, the two isolates possessed significantly higher values of chitinase activity among those tested (Table 2), a feature correlated with their entomopathogenicity [30]. Multilocus-based molecular identification of isolates *bbph2* and *bbL4* confirmed the morphology-based identification as *B*. *bassiana* s.s. Phylogenetic analyses, performed on each single marker and on the concatenated dataset, attributed the *bbph2* (isolated from the beetle *P*. *excavatus*) and *bbL4* (isolated from a soil sample) isolates to two separate *B*. *bassiana* clades: the first, with *B*. *bassiana* isolated from beetles collected in the USA (*L*. *decemlineata* and *A*. *grandis*); the second, sister of *B*. *bassiana* isolated from a weevil collected in Morocco. Even considering the limited sample size, the achieved results apparently indicate the absence of a correlation in terms of geographical origin of the isolates and host taxonomy. For example, isolates obtained from the same geographical area (Morocco) and from the same host (*Sitona discoideus*) were grouped into two separate and well-supported clades (Fig 4, S4 Table).

On the basis of the achieved results, the isolates of *B*. *bassiana*, obtained from a soil sample collected in Lattakia and from the insect *P*. *excavatus* collected in Damascus, could be considered as promising thermotolerant isolates to be used as biocontrol agents, especially against pests inhabiting Mediterranean and Subtropical areas, such as *R*. *ferrugineus* and *Tuta absoluta*. However, further studies are required to evaluate the efficiency of mass production of those isolates and their effect on non-target organisms.

In accordance with the idea that isolates’ capability to survive and grow at high temperatures is related to the specific growth habitat on regional or micro scales [17,32], the strategy adopted in this study could be considered a useful protocol for selecting thermotolerant *B*. *bassiana* isolates from high-temperature natural or semi-natural areas.

## Supporting information

S1 Fig Probit analysis for calculating the lethal concentration of *B*. *bassiana* isolates (LC_50_). Corrected mortality percentage of 3^rd^ instar larvae of *E*. *kuehinella* treated with different concentrations (log_10_) of B. bassiana isolates at the 10th day of observation.
(TIF)

S2 Fig Probit analysis for calculating the lethal time of *B*. *bassiana* isolates (LT_50_). Corrected mortality percentage of 3^rd^ instar larvae of *E*. *kuehinella* at different observation days (log_10_), after treatment with *B*. *bassiana* isolates (10^^5^ spores/ml).
(TIF)

S3 Fig Maximum likelihood tree inferred from an allignment of the ITS sequences. Branch ends with the accession number of the sequences. The scale bar at the bottom indicates the distance expressed in substitutions per site.
(TIF)

S4 Fig Maximum likelihood tree inferred from an allignment of the *Bloc* sequences. Branch ends with the accession number of the sequences. The scale bar at the bottom indicates the distance expressed in substitutions per site.
(TIF)

S5 Fig Maximum likelihood tree inferred from an allignment of the *EF1-α* sequences. Branch ends with the accession number of the sequences. The scale bar at the bottom indicates the distance expressed in substitutions per site.
(TIF)

S1 Table Isolates and sampling sites characteristics. *Beauveria bassiana* isolates and their sites of collection and source (insect host or soil sample).
(DOCX)

S2 Table Isolates agressivity evaluation using the Fungal Development Index (FDI). A scale based on the observed symptoms of *Beauveria bassiana* spores infection and development on the surface of *Ephestia kuehienella* larvae.
(DOCX)

S3 Table Molecular markers used for *B*. *bassiana* characterization. The primers, PCR amplification conditions and the references.
(DOCX)

S4 Table A list of characterized *Beauveria* isolates with their host and origin. *Beauveria isolates* used in this study as comparatives and ITS, *Bloc* and *EFα-1* GenBank accession numbers.
(DOCX)

S5 Table Biological characteristics of the obtained *Beauveria bassiana* isolates. The measured biological parameters of the obtained *B*. *bassiana* isolates at different tested temperatures.
(DOCX)

S6 Table Changes in the biological parameters of the obtained *Beauveria bassiana* isolates. Reduction percentages in the biological parameters due to the increase of tested temperatures (25°C to 30°C), and their correlation with the average temperature of the collection area.
(DOCX)
