# Supplementary figures and images for: Thermotolerant isolates of Beauveria bassiana as potential control agent of insect pest in subtropical climates
Source: PLoS One. 2019 Feb 1;14(2):e0211457. doi: 10.1371/journal.pone.0211457 (PMC6358154; doi:10.1371/journal.pone.0211457)

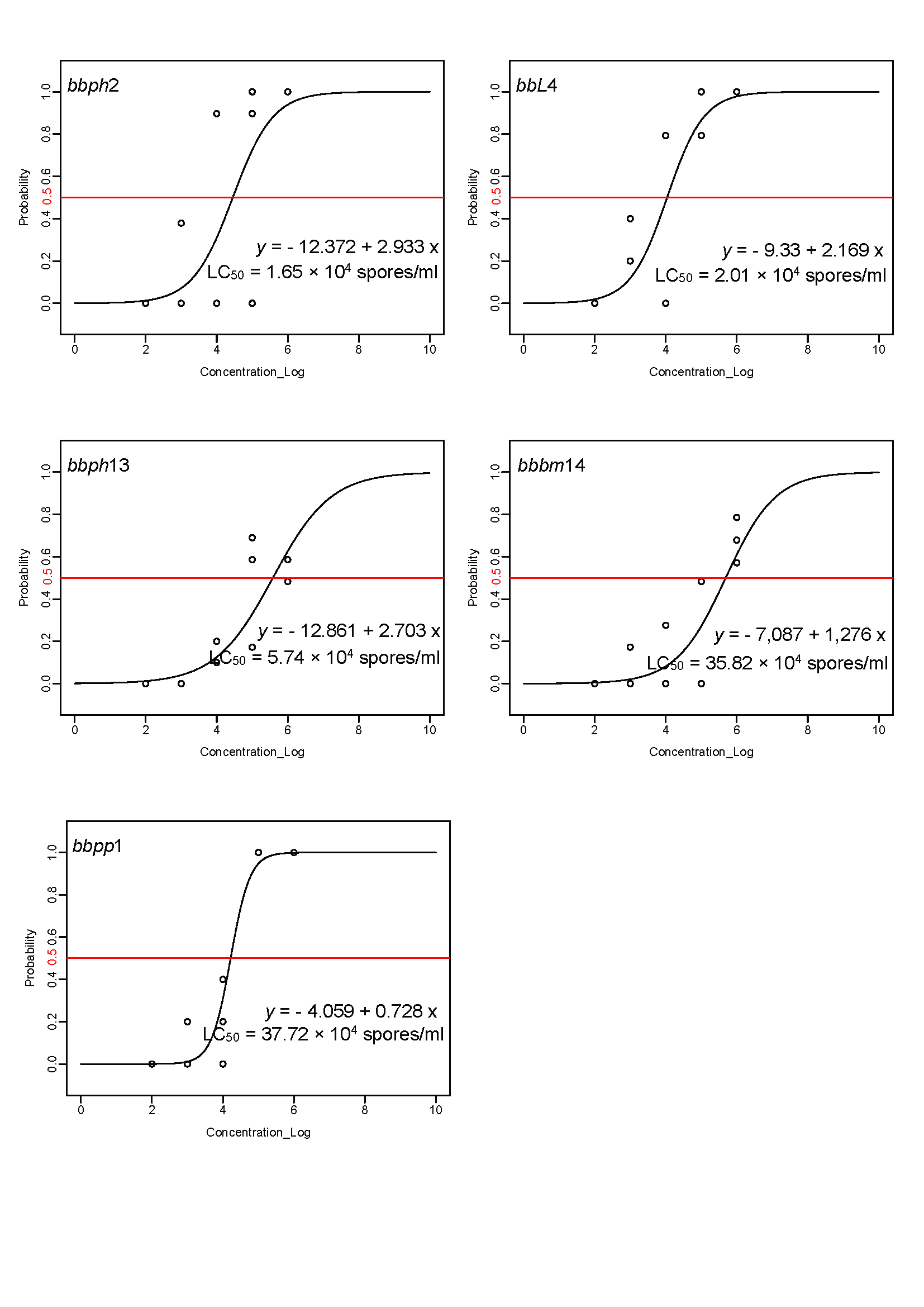

Supplement: S1 Fig — Corrected mortality percentage of 3rd instar larvae of E. kuehinella treated with different concentrations (log10) of B. bassiana isolates at the 10th day of observation. (TIF) [file pone.0211457.s001.tif]

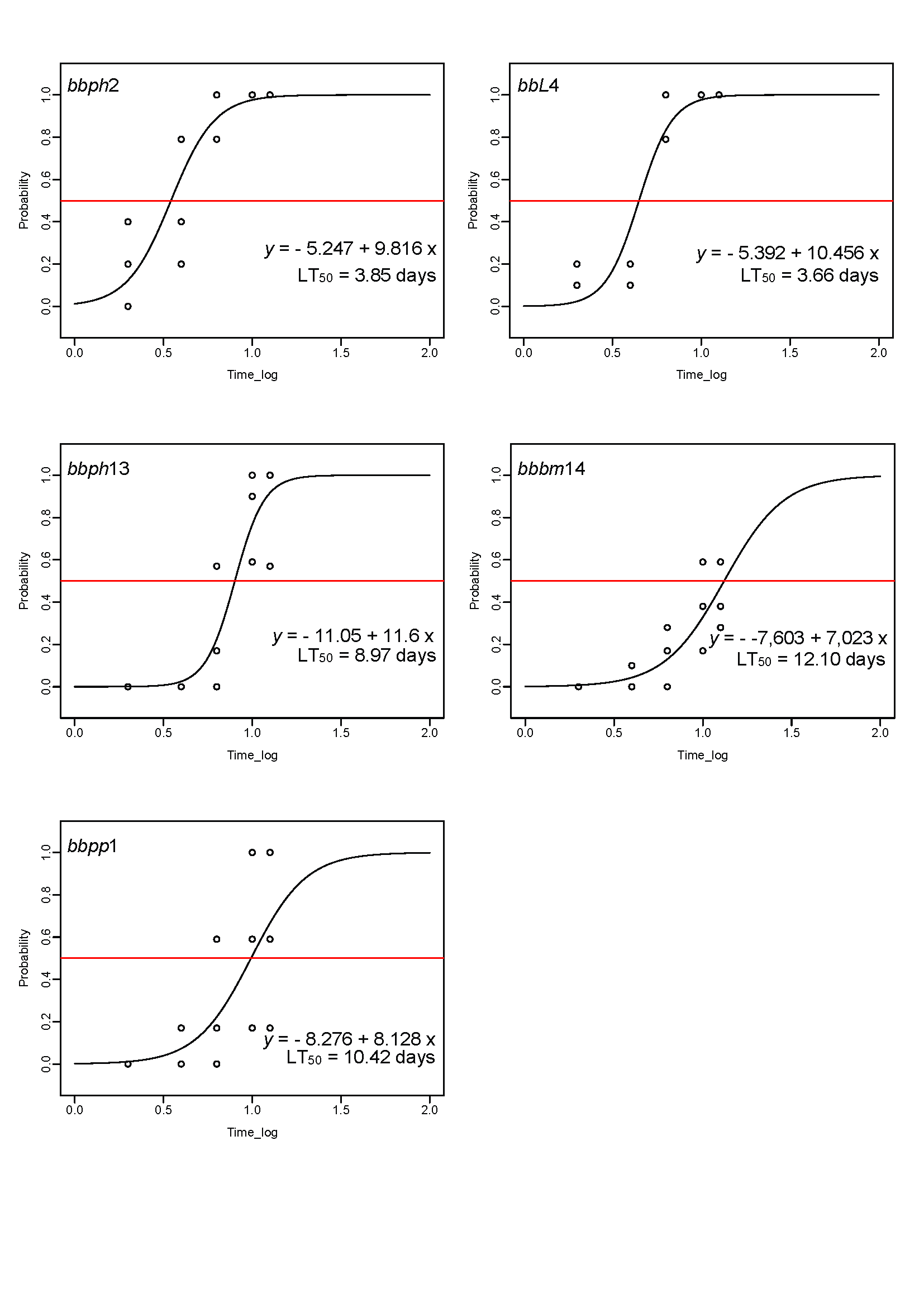

Supplement: S2 Fig — Corrected mortality percentage of 3rd instar larvae of E. kuehinella at different observation days (log10), after treatment with B. bassiana isolates (10^5 spores/ml). (TIF) [file pone.0211457.s002.tif]

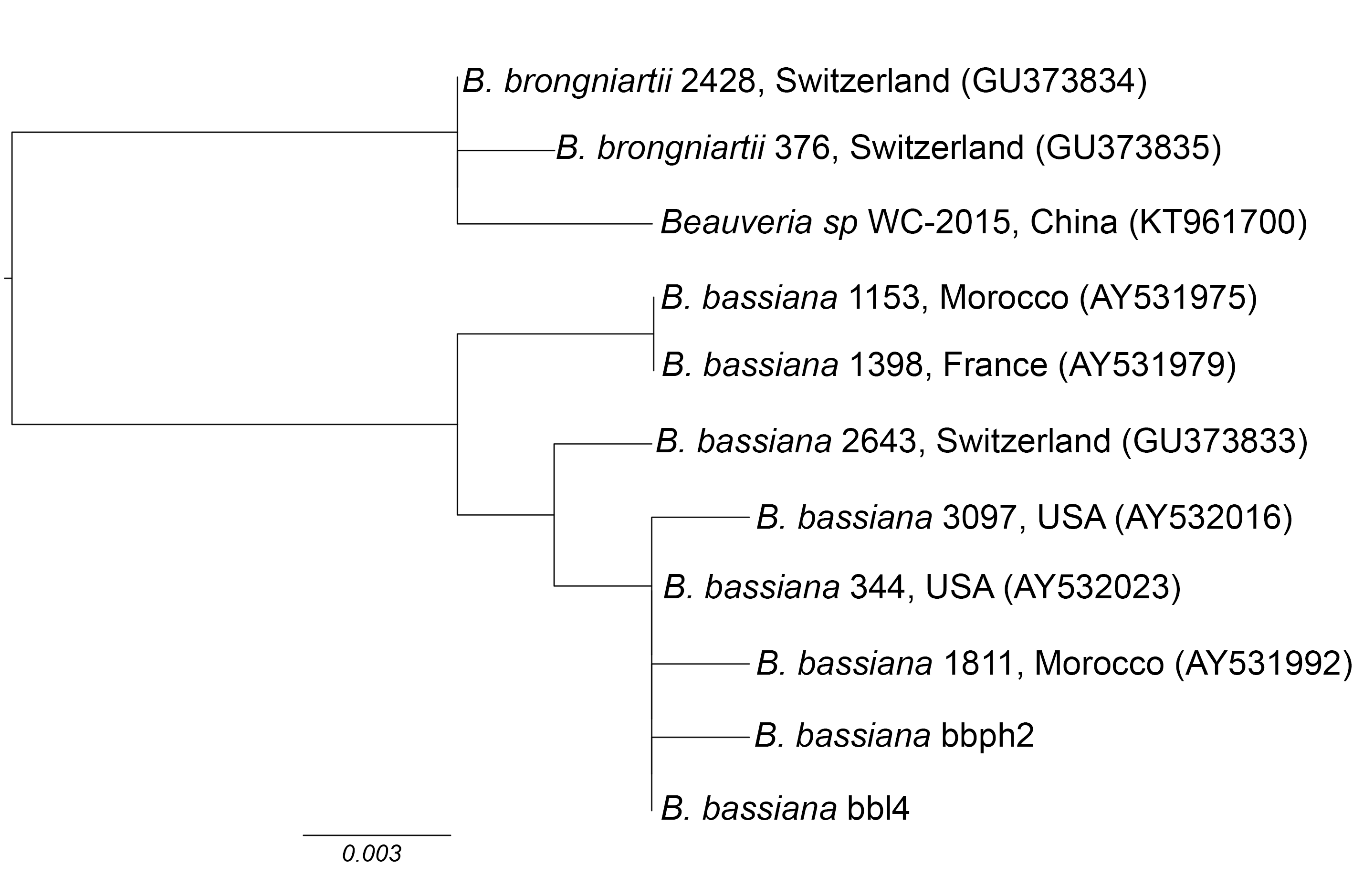

Supplement: S3 Fig — Branch ends with the accession number of the sequences. The scale bar at the bottom indicates the distance expressed in substitutions per site. (TIF) [file pone.0211457.s003.tif]

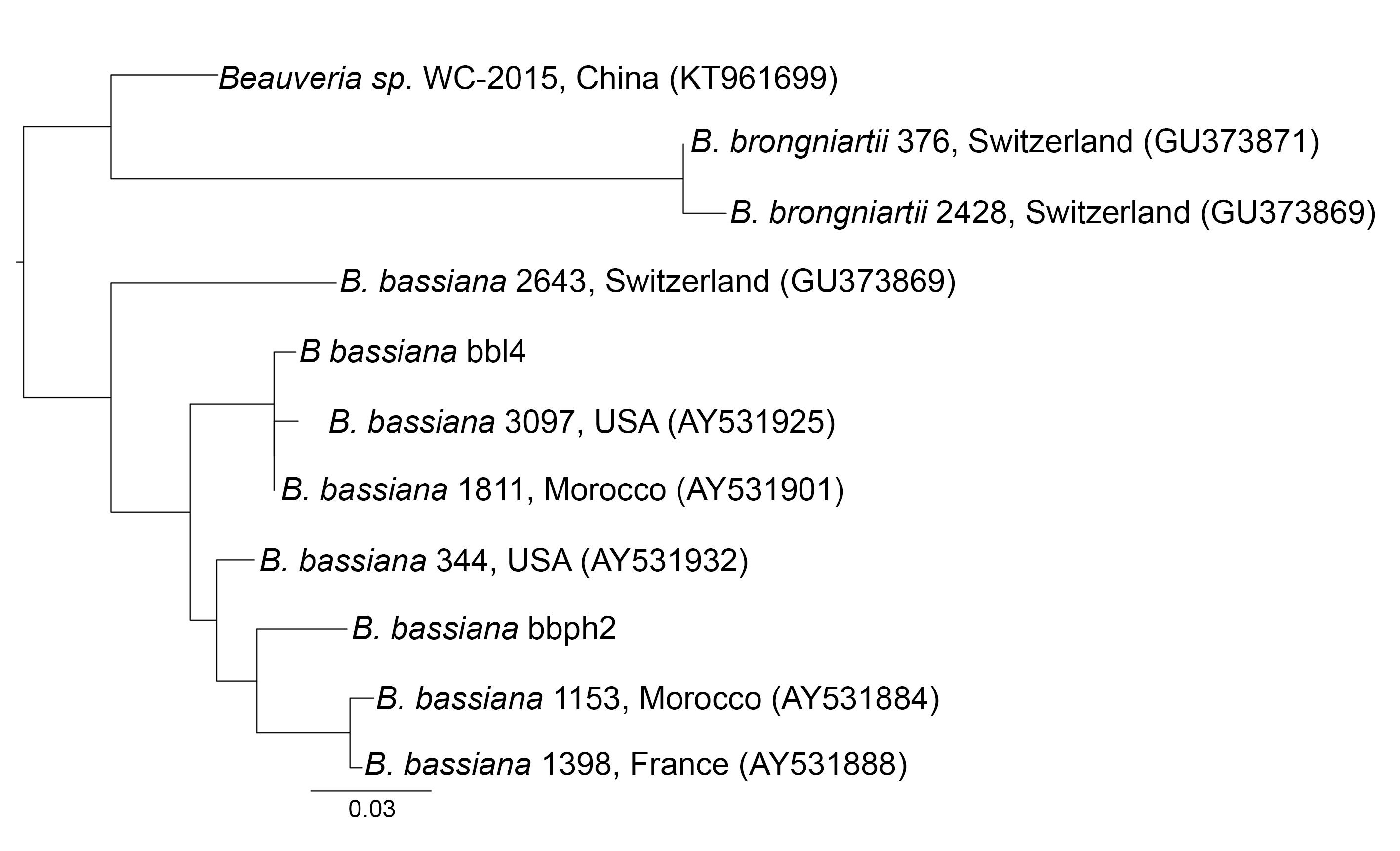

Supplement: S4 Fig — Branch ends with the accession number of the sequences. The scale bar at the bottom indicates the distance expressed in substitutions per site. (TIF) [file pone.0211457.s004.tif]

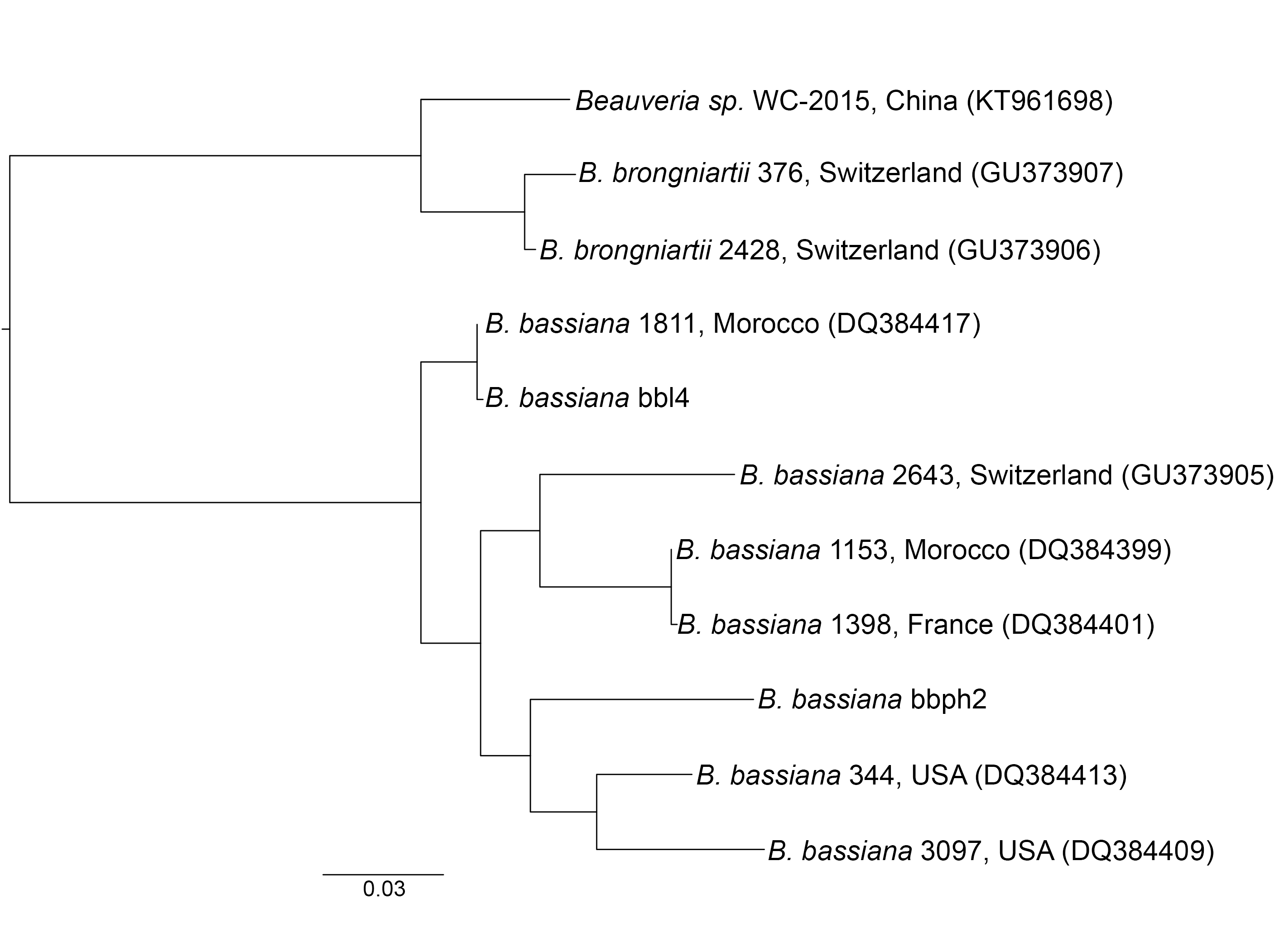

Supplement: S5 Fig — Branch ends with the accession number of the sequences. The scale bar at the bottom indicates the distance expressed in substitutions per site. (TIF) [file pone.0211457.s005.tif]
