# Supplementary material for: Thermotolerant isolates of Beauveria bassiana as potential control agent of insect pest in subtropical climates
Source: PLoS One. 2019 Feb 1;14(2):e0211457. doi: 10.1371/journal.pone.0211457 (PMC6358154; doi:10.1371/journal.pone.0211457)
Supplement: S1 Table — Beauveria bassiana isolates and their sites of collection and source (insect host or soil sample). (DOCX) [file pone.0211457.s006.docx]

**S1 Table. Isolates and sampling sites characteristics.** *Beauveria bassiana* isolates and their sites of collection and source (insect host or soil sample).

| **Isolate** | **Host** | **City** | **Latitude (N), Longitude (E)** | **Altitude (m)** | **Average temp^c^** |
| --- | --- | --- | --- | --- | --- |
| *bbpp1^a^* | *Paropta paradoxa* | Al lejat-Daraa | 32.842190°, 36.320131° | 642 | 39 |
| *bbph2* | *Phyllognathus excavatus* | Kafar Sousah-Damascus | 33.474825°, 36.267022° | 689 | 31 |
| *bbDR1^b^* | *Eurygaster integriceps* | Aleppo | - | 382 | 31 |
| *bbL4* | Soil | Lattakia | 35.536442°, 35.810572° | 67 | 27 |
| *bbca5* | *Capnodis* sp. | Hout-Al Suwayda | 32.490074°, 36.611579° | 1130 | 28 |
| *bbAr6* | Soil | Erneh-Damascus Governorate | 33.372971°, 35.884348° | 1376 | 26 |
| *bbcd7* | *Cyrambix dux* | Hama | 35.195342°, 36.649228° | 288 | 31 |
| *bbHs8* | Soil | Homs | 34.714323°, 36.761119° | 540 | 30 |
| *bbZ10* | Soil | Al Zabadani-Damascus Governorate | 33.718191°, 36.091386° | 1144 | 27 |
| *bbbm12* | *Bombix mori* | Abou Jarash-Damascus | 33.538091°, 36.318573° | 712 | 31 |
| *bbph13* | *Phyllognathus excavatus* | Al Nashabiyah-Damascus Governorate | 33.502839°, 36.488759° | 618 | 31 |
| *bbbm14* | *Bombix mori* | Abou Jarash-Damascus | 33.538091°, 36.318573° | 712 | 31 |
| *bbAr17* | Soil | Erneh-Damascus Governorate | 33.372971°, 35.884348° | 1376 | 26 |
| *bbAr18* | Soil | Erneh-Damascus Governorate | 33.372971°, 35.884348° | 1376 | 26 |
| *bbHs19* | Soil | Homs | 34.718307°, 36.752609° | 534 | 30 |
| *bbHs20* | Soil | Homs | 34.718307°, 36.752609° | 534 | 30 |
| *bbHm21* | Soil | Hama | 35.195342°, 36.649228° | 287 | 31 |
| *bbHm22* | Soil | Hama | 35.195342°, 36.649228° | 287 | 31 |
| *bbSw23* | Soil | Al Suwayda | 32.721426°, 36.589270° | 1123 | 28 |

^a^ The ID of each samples is made by appriviation of *Beauveria bassiana=* bb, appriviation of insect host or the City (pp=*Paropta paradoxa*, Hs=Homs).

^b^ This isolate was provided by ICARDA (International Center for Agricultural Research in the Dry Areas in Aleppo).

^C^ Average temperature of the hot season each year from May to late September of the sites where the sample were collected (°C) [Diebel J, Norda J. Weatherspark, Cedar lake ventures Inc. 2013].
