# Supplementary material for: Thermotolerant isolates of Beauveria bassiana as potential control agent of insect pest in subtropical climates
Source: PLoS One. 2019 Feb 1;14(2):e0211457. doi: 10.1371/journal.pone.0211457 (PMC6358154; doi:10.1371/journal.pone.0211457)
Supplement: S2 Table — A scale based on the observed symptoms of Beauveria bassiana spores infection and development on the surface of Ephestia kuehienella larvae. (DOCX) [file pone.0211457.s007.docx]

**S2 Table. Isolates agressivity evaluation using the Fungal Development Index (FDI).** A scale based on the observed symptoms of *Beauveria bassiana* spores infection and development on the surface of *Ephestia kuehienella* larvae.

| **Score** | **Observation of the fungal development in the larva** |
| --- | --- |
| 0 | living larva, no signs of infection on the body |
| 0.5 | small melanic spots on larval surface, big amount is possible |
| 1 | big melanic spots on larval surface, could join together |
| 1.5 | beginning of mycelial growth, individual hypha on softer parts of larval body |
| 2 | compact mycelium on 1/3 of the body |
| 2.5 | beginning of sporulation |
| 3 | fully sporulating mycelium on cadaver |
