# Supplementary material for: Thermotolerant isolates of Beauveria bassiana as potential control agent of insect pest in subtropical climates
Source: PLoS One. 2019 Feb 1;14(2):e0211457. doi: 10.1371/journal.pone.0211457 (PMC6358154; doi:10.1371/journal.pone.0211457)
Supplement: S3 Table — The primers, PCR amplification conditions and the references. (DOCX) [file pone.0211457.s008.docx]

**S3 Table. Molecular markers used for *B. bassiana* characterization.** The primers, PCR amplification conditions and the references.

| **Marker** | **Primers** | **Primers and PCR amplification protocol Reference** |
| --- | --- | --- |
| ITS region | ITS5 5’–GGAAGTAAAAGTCGTAACAAGG–3’  ITS4 5’–TCCTCCGCTTATTGATATGC–3’ | Rehner and Buckley 2005 |
| Bloc | B5.1F 5’–CGACCCGGCCAACTACTTTGA–3’  B3.1R 5’–GTCTTCCAGTACCACTACGCC–3’ | Rehner et al. 2006 |
| EF1-α | 983F 5’–GCYCCYGGHCAYCGTGAYTTYAT–3’  2218R 5’–ATGACACCRACRGCRACRGTYTG–3’ | Rehner et al. 2006 |

**Supporting references**

Rehner SA, Buckley EP. A *Beauveria* phylogeny inferred from nuclear ITS and *EF1-α* sequences: Evidence for cryptic diversification and links to *Cordyceps* teleomorphs. Mycologia. 2005;97:84-98.

Rehner SA, Posada F, Buckley EP, Infante F, Castillo A, Vega FE. Phylogenetic origins of African and Neotropical *Beauveria bassiana* s.l. pathogens of the coffee berry borer, *Hypothenemus hampei*. J. Invertebr. Pathol. 2006; 93: 11–21.
