# Supplementary material for: Thermotolerant isolates of Beauveria bassiana as potential control agent of insect pest in subtropical climates
Source: PLoS One. 2019 Feb 1;14(2):e0211457. doi: 10.1371/journal.pone.0211457 (PMC6358154; doi:10.1371/journal.pone.0211457)
Supplement: S4 Table — Beauveria isolates used in this study as comparatives and ITS, Bloc and EFα-1 GenBank accession numbers. (DOCX) [file pone.0211457.s009.docx]

**S4 Table. A list of characterized *Beauveria* isolates with their host and origin.** *Beauveria isolates* used in this study as comparatives and ITS, *Bloc* and *EFα-1* GenBank accession numbers.

| **Collection ID** | **Identification** | **Country** | **Host/substrate** | **Host order: family** | ***EFα-1*** | ***Bloc*** | **ITS** |
| --- | --- | --- | --- | --- | --- | --- | --- |
| 2643 | *Beauveria bassiana* | Switzerland | *Ips typographus* | Coleoptera: Scolytidae | GU373869 | GU373905 | GU373833 |
| ARSEF 1153 | *Beauveria bassiana* | Morocco | *Sitona discoideus* | Coleoptera: Curculionidae | AY531884 | DQ384399 | AY531975 |
| ARSEF 1398 | *Beauveria bassiana* | France | *Hypera postica* | Coleoptera: Curculionidae | AY531888 | DQ384401 | AY531979 |
| ARSEF 1811 | *Beauveria bassiana* | Morocco | *Sitona discoideus* | Coleoptera: Curculionidae | AY531901 | DQ384417 | AY531992 |
| ARSEF 3097 | *Beauveria bassiana* | USA | *Anthonomus grandis* | Coleoptera: Curculionidae | AY531925 | DQ384409 | AY532016 |
| ARSEF 344 | *Beauveria bassiana* | USA | *Leptinotarsa decemlineata* | Coleoptera: Chrysomelidae | AY531932 | DQ384413 | AY532023 |
| ^*^ 376 | *Beauveria brongniartii* | Switzerland | *Melolontha melolontha* | Coleoptera: Scarabaeidae | GU373871 | GU373907 | GU373835 |
| ^*^ 2428 | *Beauveria brongniartii* | Switzerland | *Meligethes aeneus* | Coleoptera: Nitidulidae | GU373870 | GU373906 | GU373834 |
| ^*^ WC-2015 | *Beauveria sp.* | China | NA | NA | KT961699 | KT961698 | KT961700 |

^*^ Isolates considered as outgroups isolates.
