# Supplementary material for: Thermotolerant isolates of Beauveria bassiana as potential control agent of insect pest in subtropical climates
Source: PLoS One. 2019 Feb 1;14(2):e0211457. doi: 10.1371/journal.pone.0211457 (PMC6358154; doi:10.1371/journal.pone.0211457)
Supplement: S5 Table — The measured biological parameters of the obtained B. bassiana isolates at different tested temperatures. (DOCX) [file pone.0211457.s010.docx]

**S5 Table. Biological characteristics of the obtained *Beauveria bassiana* isolates.** The measured biological parameters of the obtained *B. bassiana* isolates at different tested temperatures.

| **Isolate** | **Growth rate (mean ± SE)^a^** | | | **Spore production (mean ± SE) ^b^** | | | **Spore germination (mean ± SE) ^c^** | | |
| --- | --- | --- | --- | --- | --- | --- | --- | --- | --- |
|  | **20°C** | **25°C** | **30°C** | **20°C** | **25°C** | **30°C** | **20°C** | **25°C** | **30°C** |
| *bbph2* | 0.81±0.07 | 1.88±0.01 | 0.86±0.04^*^ | 1.96±0.04 | 2.98±0.06 | 2.06±0.04^*^ | 82.5±0.34^*^ | 99.27±0.23^*^ | 78.42±0.18^*^ |
| *bbph13* | 1.08±0.03 | 1.83±0.15 | 0.81±0.03^*^ | 1.92±0.03 | 3.9±0.1^*^ | 2.15±0.05^*^ | 80.87±0.39 | 98.17±0.52^*^ | 73.63±0.39^*^ |
| *bbpp1* | 0.88±0.01 | 1.86±0.06 | 0.74±0.1 | 2.01±0.06 | 2.87±0.07 | 1.52±0.03 | 80.04±0.45 | 96.7±0.3 | 70.59±0.22^*^ |
| *bbL4* | 1.18±0.05^*^ | 2.19±0.07^*^ | 0.79±0.05^*^ | 2.52±0.26^*^ | 3.46±0.05^*^ | 2±0.03^*^ | 77.06±0.89 | 97.33±0.4^*^ | 70.08±0.29^*^ |
| *bbbm14* | 0.99±0.01 | 1.89±0.07 | 0.69±0.04 | 1.88±0.06 | 2.6±0.05 | 1.22±0.03 | 81.4±0.56 | 98.44±0.45^*^ | 69.95±0.3^*^ |
| *bbDR1* | 1.01±0.06 | 2.14±0.05^*^ | 0.66±0.03 | 1.81±0.04 | 2.74±0.01 | 1.13±0.01 | 82.71±0.51 | 92.73±0.24 | 63.77±0.17 |
| *bbbm12* | 1.11±0.03 | 1.71±0.08 | 0.6±0.03 | 2.01±0.07 | 2.51±0.03 | 1.03±0.01 | 73.86±0.65 | 98.5±0.24^*^ | 64.03±0.16 |
| *bbZ10* | 1.19±0.04^*^ | 2.13±0.09^*^ | 0.54±0.04 | 1.58±0.03 | 1.71±0.04 | 0.77±0.02 | 84.72±0.48 | 97.1±0.29 | 63.12±0.19 |
| *bbHs20* | 0.91±0.03 | 1.84±0.17 | 0.65±0.03 | 1.17±0.05 | 1.74±0.02 | 1.04±0.02 | 88.82±0.76 | 97.73±0.38^*^ | 62.55±0.25 |
| *bbHm22* | 0.97±0.03 | 1.94±0.1 | 0.61±0.04 | 1.27±0.04 | 2.43±0.02 | 1.07±0.01 | 84.07±0.46 | 97.7±0.37^*^ | 62.53±0.23 |
| *bbHs19* | 0.89±0.02 | 1.87±0.09 | 0.55±0.05 | 1.96±0.04 | 2.14±0.06 | 0.92±0.03 | 89.85±0.6^*^ | 97.93±0.42^*^ | 61.7±0.26 |
| *bbHm21* | 0.85±0.03 | 1.95±0.06 | 0.55±0.03 | 1.4±0.06 | 2.31±0.02 | 0.99±0.01 | 89.38±0.8^*^ | 97.57±0.29 ^*^ | 61.47±0.18 |
| *bbcd7* | 1.04±0.02 | 2.04±0.07 | 0.67±0.03 | 1.74±0.03 | 2.7±0.02 | 1.13±0.01 | 56.24±0.31 | 96.47±0.48 | 59.81±0.3 |
| *bbHs8* | 0.84±0.04 | 1.94±0.05 | 0.64±0.03 | 1.67±0.01 | 1.79±0.01 | 0.73±0.01 | 78.94±0.43 | 96.47±0.25 | 59.81±0.15 |
| *bbca5* | 1.26±0.05^*^ | 2.24±0.12^*^ | 0.51±0.03 | 1.6±0.01 | 1.85±0.04 | 0.77±0.01 | 77.84±0.76 | 97.8±0.27^*^ | 58.68±0.16 |
| *bbAr6* | 1.22±0.05^*^ | 2.17±0.12^*^ | 0.44±0.03 | 1.59±0.02 | 2.04±0.03 | 0.79±0.01 | 80.68±0.59 | 97.27±0.51^*^ | 57.39±0.3 |
| *bbAr18* | 1.21±0.05^*^ | 2.04±0.07 | 0.47±0.05 | 1.46±0.03 | 1.64±0.03 | 0.71±0.01 | 90.79±0.5^*^ | 96.6±0.23 | 55.06±0.13 |
| *bbAr17* | 1.21±0.09^*^ | 2.21±0.14^*^ | 0.44±0.05 | 1.2±0.04 | 1.57±0.02 | 0.56±0.01 | 90.32 ±0.48^*^ | 96.27±0.26 | 53.91±0.14 |
| *bbSw23* | 1.11±0.07 | 2.2±0.05^*^ | 0.46±0.03 | 0.95±0.1 | 1.19±0.03 | 0.45±0.01 | 86.04±0.69 | 97.47±0.31^*^ | 56.53±0.18 |
| F^d^ | 13.72 | 3.355 | 9.134 | 134.66 | 291.01 | 513.18 | 63.45 | 12.5 | 795.46 |
| Sig. | 0.000 | 0.000 | 0.000 | 0.000 | 0.000 | 0.000 | 0.000 | 0.000 | 0.000 |

^a^ Growth rate in mm/day.

^b^ Spore production in spores/ml.

^c^ Spore germination as percentage of the germinated spores in 300 observed spores (%).

^d^ Calculated Fisher’s test value.

^*^ In the same column indicate the significantly higher values (α=0.05).

At the growth temperature of 35°C, no growth was recorded.
