# Supplementary material for: Thermotolerant isolates of Beauveria bassiana as potential control agent of insect pest in subtropical climates
Source: PLoS One. 2019 Feb 1;14(2):e0211457. doi: 10.1371/journal.pone.0211457 (PMC6358154; doi:10.1371/journal.pone.0211457)
Supplement: S6 Table — Reduction percentages in the biological parameters due to the increase of tested temperatures (25°C to 30°C), and their correlation with the average temperature of the collection area. (DOCX) [file pone.0211457.s011.docx]

**S6 Table. Changes in the biological parameters of the obtained *Beauveria bassiana* isolates.** Reduction percentages in the biological parameters due to the increase of tested temperatures (25°C to 30°C), and their correlation with the average temperature of the collection area.

| **Isolates** | **Growth rate reduction (%)** | **Spore production reduction (%)** | **Spore germination reduction (%)** | **Average temperature^a^** |
| --- | --- | --- | --- | --- |
| *bbph2* | 54.25 ± 1.84 ^b^ | 30.84 ± 1.91 | 21 ± 0.26 | 31 |
| *bbph13* | 55.5 ± 3.73 | 42.18 ± 1.2 | 25 ± 0.56 | 31 |
| *bbpp1* | 60.13 ± 4.91 | 44.84 ± 1.88 | 27 ± 0.32 | 39 |
| *bbbm14* | 63.43 ± 2.42 | 47.01 ± 1.64 | 28 ± 0.42 | 31 |
| *bbL4* | 63.88 ± 2.43 | 53.06 ± 1.44 | 28.94 ± 0.44 | 27 |
| *bbHs20* | 64.43 ± 3.44 | 54.47 ± 1.35 | 34.33 ± 1.06 | 30 |
| *bbbm12* | 65.64 ± 2.18 | 54.95 ± 1.55 | 34.99 ± 0.28 | 31 |
| *bbHs8* | 66.98 ± 1.69 | 54.97 ± 0.66 | 34.99 ± 0.23 | 30 |
| *bbcd7* | 67.11 ± 1.81 | 55.96 ± 0.55 | 36 ± 0.36 | 31 |
| *bbHm22* | 68.47 ± 2.54 | 56.69 ± 0.99 | 36 ± 0.34 | 31 |
| *bbDR1* | 69.14 ± 1.51 | 56.97 ± 1.82 | 36.99 ± 0.38 | 31 |
| *bbHs19* | 70.5 ± 2.86 | 57.14 ± 0.57 | 37 ± 0.26 | 30 |
| *bbHm21* | 71.76 ± 1.69 | 57.81 ± 0.4 | 38 ± 0.44 | 31 |
| *bbZ10* | 74.59 ± 2.06 | 58.04 ± 0.61 | 38 ± 0.22 | 27 |
| *bbAr18* | 76.92 ± 2.34 | 58.15 ± 0.48 | 40 ± 0.23 | 26 |
| *bbca5* | 77.17 ± 1.76 | 58.36 ± 1.02 | 41 ± 0.44 | 28 |
| *bbSw23* | 79.07 ± 1.33 | 61.27 ± 0.74 | 42 ± 0.26 | 28 |
| *bbAr6* | 79.66 ± 1.73 | 62.16 ± 1.25 | 43 ± 0.19 | 26 |
| *bbAr17* | 79.99 ± 2.44 | 64.32 ± 0.77 | 44 ± 0.21 | 26 |
| Pearson correlation (r) | -0.564 ^*^ | -0.453 ^*^ | -0.544 ^*^ |  |
| Sig. | P-value < 0.001 | P-value < 0.001 | P-value < 0.001 |  |

^a^ Annual average temperature of the hot season each year from May to late September (weatherspark.com) of the sites where the sample were collected (°C).

^b^ Values in mean ± SE.

^*^ Significant at α=0.05.
